# Pediatric endocardial temporary pacemaker implantation: Clinical characteristics and outcomes from a Chinese National Regional Health Center

**DOI:** 10.1002/pdi3.2508

**Published:** 2024-11-18

**Authors:** Zhongyuan Liu, Kaijun Zhang, Jun Ren, Penghui Yang, Mi Li, Ping Xiang

**Affiliations:** ^1^ Department of Cardiology Children's Hospital of Chongqing Medical University National Clinical Research Center for Child Health and Disorders Ministry of Education Key Laboratory of Child Development and Disorders National Clinical Key Cardiovascular Specialty Chongqing China

**Keywords:** children, endocardial temporary pacemakers, national regional medical center

## Abstract

Endocardial temporary pacemakers (TP) are widely used in the treatment of cardiovascular diseases in adults, but their application and clinical experience in the treatment of pediatric cardiovascular cases are relatively limited. This study aims to share the experience of using endocardial TP at a pediatric medical center in China. The study included patients who received their first endocardial TP implantation in the Department of Cardiology at Children's Hospital of Chongqing Medical University, China, from 2016 to 2022. A retrospective analysis of their clinical data was conducted. Our study involved 45 children who underwent endocardial TP implantation. Among these, 25 children (55.56%) converted to sinus rhythm after pacemaker parameter adjustment and the pacemaker was successfully removed; 13 children (28.89%) did not recover sinus rhythm and ultimately required a permanent pacemaker; in 4 cases, the family refused permanent pacemaker implantation; in 1 case, the treatment was abandoned by the family; and 2 children died during hospitalization. Notably, among the 20 children with fulminant myocarditis (FM), 16 children (80.00%) converted to sinus rhythm after pacemaker parameter adjustment and the pacemaker was successfully removed; 3 children (15.00%) ultimately received a permanent pacemaker; and 1 child died during hospitalization. Endocardial TP have shown remarkable efficacy in the treatment of critical heart disease, especially FM.

## Graphical abstract

Endocardial TP have shown remarkable efficacy in the treatment of critical heart disease, especially fulminant myocarditis.

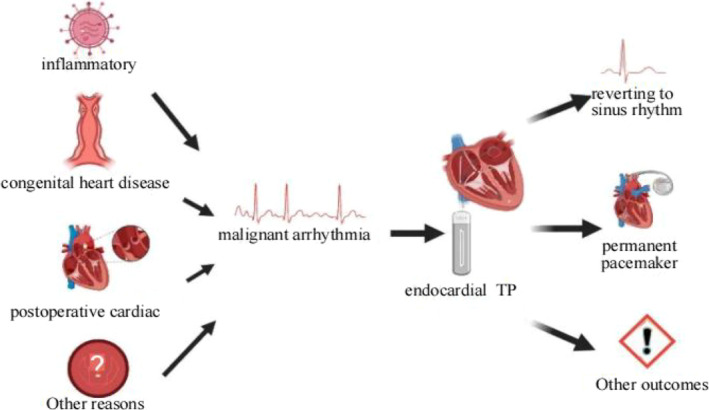

## 1 INTRODUCTION

A cardiac pacemaker is a medical device that employs low‐energy electrical pulses to mimic the electrophysiological function of the heart muscle, thereby regulating heart rate and cardiac output to address various cardiac dysfunctions.^1^ Since the first permanent pacemaker implantation in 1958, indications for pacemaker therapy have significantly expanded to encompass a wide range of cardiac diseases. These include bradycardic arrhythmias (such as atrioventricular block), tachyarrhythmias (such as idiopathic ventricular tachycardia), cardiac arrest, sinoatrial node disease, acute myocardial infarction, specific types of syncope, advanced heart failure, permanent pacemaker failure, as well as prophylactic or perioperative use. Pacemaker technology has evolved from the initial single‐chamber fixed‐rate devices to modern multi‐chamber variable‐rate devices. These advanced devices not only perform pacing but also provide cardioversion and defibrillation to better support physiological needs.^2^, ^3^ Clinical studies have shown that these advanced implantable pacemakers significantly improve cardiac function in patients with malignant arrhythmias from various etiologies, greatly enhancing patient survival rates.^4^

Endocardial temporary pacemakers (TP) typically involves the non‐permanent placement of a cardiac pacemaker into the right ventricle via the venous system, commonly through the femoral vein. The duration when the pacing electrode is placed usually ranges from 1 to 2 weeks, and rarely exceeds 1 month. The device is immediately removed upon achieving the diagnostic or therapeutic objective.^5^ Despite potential complications associated with endocardial TP therapy, its ease of use, minimal invasiveness, rapid effectiveness, and stable outcomes have led to its widespread use in the treatment of various acute and critical cardiovascular diseases, such as fulminant myocarditis (FM) and malignant arrhythmias, significantly improving the success rate of emergency treatment and patient prognosis. As a transitional and protective measure, TP plays a crucial role in enhancing cardiac output, stabilizing hemodynamics, and improving myocardial perfusion, thereby aiding the recovery of damaged cardiac muscle. Thus, it is widely utilized in treating cardiovascular diseases.^6^ In recent years, with improved reliability of pacing systems, advancements in pacing technology, and accumulation of clinical experience, TP has been increasingly applied in the treatment of acute severe cardiovascular diseases in children, especially in treating FM, malignant arrhythmias, and postoperative arrhythmias in congenital heart disease.^7^, ^8^ Compared to adults, however, data on the use of TP in children remain relatively limited.^9^ This study included patients who underwent their first TP implantation in the Department of Cardiology at Children's Hospital of Chongqing Medical University, China, with a retrospective analysis conducted on their clinical data. The study aimed to assess the clinical characteristics and outcomes of pediatric patients undergoing TP implantation at this medical center.

## 2 METHODS

### 2.1 Patients

This study retrospectively analyzed pediatric patients who underwent their first endocardial temporary cardiac pacemaker implantation at the Department of Cardiology of Children's Hospital of Chongqing Medical University (a Chinese National Regional Health Center), China, from 2016 to 2022. The inclusion criterion was children who met the indications for endocardial TP implantation, while exclusion criteria  comprised patients who received epicardial TP implantation and those whose family refused the endocardial TP implantation despite clinical indications.

The data collected in this study included demographic information (gender, age, weight), description of the condition upon admission (clinical symptoms, duration of symptoms, primary admission diagnosis), information related to pacemaker implantation (implantation and removal times, duration of operation, pacing mode, device operating duration), key examinations and test results before and after implantation (including echocardiography, electrocardiogram, cardiac enzyme spectrum analysis), as well as the clinical outcomes of the children (including changes in condition during hospitalization, recurrence of symptoms, survival status), and follow‐up data (including survival status, electrocardiogram, and echocardiography results).

In this study, all legal guardians of the children were fully informed about the potential risks and complications associated with the endocardial TP implantation prior to the operation and had signed informed consent forms. The study adhered to the principles of the Declaration of Helsinki (revised version 2018) and was approved by the Ethics Committee of Children's Hospital of Chongqing Medical University.

### 2.2 Endocardial TP implantation procedure

The implantation of the endocardial TP was strictly performed in accordance with the relevant medical guidelines.^10^ Under general anesthesia, the child was placed in a supine position. The surgical site was routinely disinfected and draped with a sterile towel. A venous sheath was inserted through a puncture in the right femoral vein. Using an appropriate catheter and guided by a guidewire, a pathway was established from the right femoral vein to the inferior vena cava, right atrium, and right ventricle, and the pacing electrode was advanced to the apex of the right ventricle. Based on electrocardiographic data, the parameters of the endocardial TP (including heart rate, output voltage, and sensing voltage) were set. The pacemaker electrode was fixed with tensioned skin sutures, and the surgical site was then bandaged. The pacemaker setting and the duration of its placement were comprehensively assessed and adjusted by the attending physician according to the child's clinical condition.

Type of Temporary Pacemaker: EV4543 miniature temporary cardiac pacemaker, Pace Medical. Inc., Waltham, MA, USA.

### 2.3 Statistical analysis

Continuous variables following a normal distribution were expressed as mean ± standard deviation, while non‐normally distributed variables were represented by the median and interquartile range. Group comparisons were made using the Mann–Whitney U non‐parametric test. All statistical analyses were conducted using SPSS 26.0 software (IBM Corporation, Armonk, New York, NY, USA). A *p*‐value of less than 0.05 was considered statistically significant.

## 3 RESULTS

### 3.1 Statistical characteristics of the population

A total of 45 children underwent endocardial TP implantation at our center, including 23 males and 22 females. Ages at the time of operation ranged from 8 to 194 months, with an average age of 96.80 ± 42.18 months. Weights ranged from 7 to 70 kg, with an average weight of 27.16 ± 12.95 kg. Among these children, 20 were diagnosed with FM, including 12 males and 8 females, with ages ranging from 18 to 166 months at the time of operation, and an average age of 98.05 ± 37.53 months. Their weights ranged from 8 to 53 kg, with an average weight of 24.53 ± 9.67 kg.

### 3.2 Clinical features prior to endocardial TP placement

Based on the chief complaints and medical histories, the primary clinical symptoms of the 45 children included syncope (12 cases), convulsions or consciousness disturbances (14 cases), fever, vomiting, and abdominal pain (15 cases), discomfort in the precordial area or fatigue (7 cases), and arrhythmias detected through examinations (5 cases). Each child may have exhibited one or multiple clinical symptoms.

According to the classification of indications for pacemaker implantation in the ESC Guidelines for Cardiac Pacing and Cardiac Resynchronisation Therapy 2021, we catagorized the cases as follows: 24 with asystole, 37 with complete atrioventricular blocks (CAVB), 3 with sick sinus syndromes, 8 with postoperative congenital heart diseases, and 2 with long QT syndromes. These data were compiled on the basis of individual diagnoses, and each child may have one or more of the above conditions.

Notably, among the 45 children, 20 were diagnosed with FM, all accompanied by CAVB, 10 were complicated with asystole, 12 with cardiogenic shock, and 4 with severe pneumonia. The above data were compiled based on individual diseases, and each child could have one or multiple comorbidities.

### 3.3 The use of endocardial TP

All 45 children successfully underwent the implantation of the endocardial TP implantation. The average duration of the procedure was 41.50 ± 16.74 min (ranging from 15 to 85 min). The pacemakers were set to ventricular pacing, ventricular sensing, inhibition (VVI) mode, with an average initial sensing voltage of 1.49 ± 0.56 mV, output voltage of 1.76 ± 1.18 V, and pacing rate averaging 75.00 ± 12.08 beats per minute.

Detailed data for the 20 children diagnosed with FM are presented in Table 1. The median time from onset of illness to successful implantation of the endocardial TP was 51.47 h (ranging from 24.75 to 154.02 h), and the median time from admission to successful pacemaker implantation was 3 h (ranging form 0.75 to 36.28 h). The median operating time of the temporary pacemaker was 111.60 h (ranging from 8 to 494.92 h). The median time from cessation of the endocardial TP to its removal was 3 days (ranging from 2 to 9 days), and the median hospital stay was 19.25 days (ranging from 8.29 to 30.57 days).

**TABLE 1:** Application of endocardial TP implantation in 20 children with FM.

No.	Time onset‐surgery (h)	Time admission‐surgery (h)	Time working (d)	Time stop‐removal (d)	Time of hospitalization (d)	Outcome
1	25.72	1.72	6.21	–	14.94	Permanent Pacemaker
2	35.47	11.47	4.89	–	26.03	Permanent Pacemaker
3	154.02	10.02	20.62	–	25.55	Permanent Pacemaker
4	25.10	1.10	3.65	2	10.74	Fine
5	49.93	1.93	1.58	2	8.29	Fine
6	51.17	3.17	0.33	2	18.08	Fine
7	25.62	1.62	1.58	9	28.38	Fine
8	51.47	3.47	11.57	3	17.31	Fine
9	74.30	2.30	2.35	4	15.16	Fine
10	86.92	14.92	4.45	3	27.21	Fine
11	24.75	0.75	6.94	2	14.23	Fine
12	79.67	7.67	4.20	7	20.89	Fine
13	146.83	2.83	4.77	2	22.76	Fine
14	49.22	1.22	6.85	2	20.64	Fine
15	73.90	1.90	10.54	3	19.26	Fine
16	84.28	36.28	4.53	5	20.28	Fine
17	52.98	4.98	19.39	3	30.57	Fine
18	66.40	18.40	1.58	2	14.46	Fine
19	50.28	2.28	3.70	4	14.13	Fine
20	25.72	1.72	11.35	–	21.11	Not cured

*Note*: Time onset‐surgery (h): time from onset of symptoms to TP implantation (hours). Time admission‐surgery (h): Time from hospital admission to TP implantation (hours). Time working (d): duration of TP working (days). time stop‐removal (d): time from stopping to removal of TP (days). Time of hospitalization (d): duration of hospitalization (days). TP: temporary pacemaker.

### 3.4 Clinical outcomes after endocardial TP placement

Among the 45 children who successfully underwent endocardial TP implantation, 25 children (55.56%, including 17 with FM) had their heart rhythm converted to sinus rhythm and successfully removed the pacemaker after adjusting its settings. Thirteen children (28.89%) did not recover sinus rhythm after adjustment or discontinuation of the endocardial TP and ultimately required a permanent pacemaker. The families of 4 children refused the implantation of a permanent pacemaker (these children had no significant clinical symptoms, and the need for a pacemaker was discovered during a physical examination). One child's family opted to discontinue treatment, and two children died during hospitalization. During the hospital stay, only one child required emergency adjustment due to pacemaker electrode displacement. No pacing disturbances, myocardial perforations, cardiac tamponade, pneumothorax, infections, or puncture‐related complications (such as subcutaneous hematomas, venous thrombosis) were observed in the remaining children.

Among the 20 children diagnosed with FM, 16 (80.00%) successfully converted to sinus rhythm after adjusting endocardial TP parameters, allowing for seccessful removal of the device. Three children (15.00%) did not recover sinus rhythm after attempts to adjust or turn off the endocardial TP and ultimately required a permanent pacemaker. One child died during hospitalization (as shown in Table 1). The comparison of biochemical indicators before and after the implantation (results of the last examination before the endocardial TP was turned off) in 16 children who successfully reverted to sinus rhythm showed significant decreases in heart rate, creatine kinase‐MB, myoglobin, hypersensitive troponin I, and B‐type natriuretic peptide (as shown in Table 2). The echocardiograms at discharge showed an average ejection fraction (EF, %) of 69.20 ± 5.47 and an average fractional shortening (FS, %) of 38.27 ± 4.60.

**TABLE 2:** Fulminant myocarditis myocardial enzyme profile in the 16 children who successfully reverted to sinus rhythm with FM.

Parameters	Preoperative	Postoperative	*p*‐value
Creatine kinase‐MB isoenzyme ((ug/L))	25.06 (0, 78.39)	1.85 (0, 21.13)	0.0001
Myoglobin (ug/L)	85.22 (13.55, 1297.40)	18.05 (7.29, 435.86)	0.0002
High‐sensitivity troponin I (ug/L)	9.07 (0, 78.70)	0.39 (0, 4.73)	0.0038
Brain natriuretic peptide (pg/ml)	276.95 (27.34, 2296.37)	56.5 (6.22, 1759.12)	0.0096

### 3.5 Follow‐up outcome

The median follow‐up time after discharge was 4.3 years (ranging from 0.8 to 10.2 years), and no cases of valvular regurgitation or cardiac enlargement were found during our follow‐up. As of the last follow‐up, there were no additional deaths except for the one child whose treatment was abandoned. Children who were treated with TP and successfully converted to normal sinus rhythm did not exhibit any of the previous clinical symptoms during the follow‐up period. Those who did not convert to normal sinus rhythm after TP treatment and ultimately received a permanent pacemaker also did not experience similar clinical symptoms during the limited follow‐up period. Focused follow‐ups were conducted for 4 children for whom TP treatment was ineffective and who met the criteria for permanent pacemaker implantation but refused it. These children are all alive but occasionally experience symptoms of chest tightness and fatigue. Electrocardiogram results were the same as before, and echocardiography showed normal cardiac function.

## 4 DISCUSSION

As a transitional and protective measure, endocardial TP effectively improves cardiac output, stabilize hemodynamics, and enhances myocardial perfusion, aiding in the recovery of diseased cardiac muscle.^11^ The indications for endocardial TP use in children are similar to those in adults, with the most common situations being FM combined with CAVB and various arrhythmias resulting in asystole syndrome. Despite continuous advancements in pacing technology and increased clinical experience, the clinical experience with endocardial TP implantation in children remains relatively limited.

In this retrospective study, we share the single‐center experience of endocardial TP use in 45 children over the past seven years. Syncope was the most common clinical manifestation leading to endocardial TP implantation, while FM combined with CAVB and asystole syndrome were the most common clinical diagnoses. Of the 45 patients who underwent endocardial TP implantation, 25 (55.56%) had their heart rhythm converted to normal sinus rhythm and successfully had the pacemaker removed after adjusting its settings, while13 children (28.89%) did not recover normal sinus rhythm and ultimately required a permanent pacemaker. This reflects the clinical characteristics of endocardial TP therapy, which is primarily used for symptomatic bradycardia and various reversible cardiac conduction blocks. Additionally, endocardial TP can serve as a bridge to permanent cardiac pacing when neither the criteria for permanent pacemaker implantation are met nor implantation is feasible.

CAVB is the most common condition requiring endocardial TP implantation, with most CAVB patients ultimately needing a permanent pacemaker.^5^ In this study, 37 out of 45 children (82.22%) were diagnosed with CAVB. Of these 37 children with CAVB, 12 (32.43%) eventually required a permanent pacemaker, which is significantly lower than the previously reported 64.2%.^5^ Notably, in our study, the proportion of patients with CAVB caused by FM who eventually required a permanent pacemaker was not high. At our medical center, 37 children were admitted with CAVB, and 20 of them had FM. All these children successfully underwent endocardial TP implantation, but only 3 children (15.00%) eventually required a permanent pacemaker. This suggests that using a endocardial TP as a transitional treatment for CAVB caused by FM is effective. We believe that in treating CAVB caused by FM, endocardial TP can effectively reverse CAVB to sinus rhythm. This not only shortens the critical period and reduces the mortality rate during the acute phase but also mitigates the reduction in quality of life and financial burden on the child and their family due to the implantation of a permanent pacemaker.

FM in children often presents with a rapid and aggressive onset, with early manifestations being primarily extracardiac, making diagnosis challenging. Within the first 24–48 h of onset, various arrhythmias, asystole syndrome, heart failure, cardiogenic shock, and even sudden cardiac death may occur. Therefore, FM is the most common critical condition requiring the use of endocardial TP.^12^, ^13^ Early implantation of endocardial TP can provide cardiac pump function support, which is of significant clinical importance for improving both short‐term and long‐term prognosis.^14^ In this study, a total of 45 patients underwent endocardial TP treatment, and 20 (44.44%) of them were treated for FM. Among these 20 FM patients, 16 (80.00%) patients successfully converted to sinus rhythm after adjusting the pacemaker settings and eventually had the device removed. Considering the significant improvement in cardiac enzyme spectrum and the normal levels of EF and FS shown in echocardiograms in these 16 children post‐TP implantation, it is evident that endocardial TP treatment can effectively improve cardiac output and hemodynamics, enhance myocardial perfusion, and facilitate the recovery of the diseased myocardium, thereby significantly reducing mortality and improving prognosis in children with FM. At our medical center, endocardial TP have been widely used in the treatment of children with FM, playing a vital role in saving lives and improving prognosis. This achievement is closely related to the efficient clinical handling of FM patients by our cardiology department. Our study shows that children with FM can receive a prompt and accurate diagnosis and aggressive treatment upon admission. The median time from onset to successful endocardial TP implantation was 51.47 h (ranging from 24.75 to 154.02 h), while the median time from admission to successful endocardial TP implantation was only 3 h (ranging from 0.75 to 36.28 h).

Since the first successful use of transvenous temporary pacing therapy was reported in 1967, the basic concept of temporary pacing therapy has not significantly changed despite the risks associated with surgery‐related complication.^14^ Compared to adults, endocardial TP implantation in children faces more challenges in terms of indications determination, pacemaker parameter settings, and operative techniques. However, in this study, all children successfully underwent endocardial TP implantation via the right femoral vein route, achieving a technical success rate of 100%. There were no occurrences of pacing disturbances, myocardial perforation, cardiac tamponade, pneumothorax, infection, or puncture‐related complications (such as subcutaneous hematomas, venous thrombosis) during hospitalization, with only one child requiring emergency adjustment due to pacemaker electrode displacement. The low complication rate of endocardial TP treatment at our medical center may be attributed to our rigorous medical system, clinical skills, and comprehensive postoperative care. Although patients undergoing transvenous temporary cardiac pacing face a higher risk of surgery‐related complications,^10^ our clinical application is limited to emergency treatment of patients with severe bradycardia causing syncope and/or hemodynamic impairment. Due to the instability of endocardial TP leads, we minimize the duration of endocardial TP operation until either the rhythm successfully converts to sinus rhythm or a more permanent treatment plan is established. Comprehensive clinical care, including regular changing of puncture site dressings, maintaining dry dressings to prevent infection, and moderate activity of the child to prevent thrombosis, is crucial for the success of endocardial TP treatment.

Currently, there is no consensus in the medical community regarding the optimal timing for the removal of endocardial TP. In this study, the analysis of patients with FM showed that the median operating time of the endocardial TP was 111.6 h (ranging from 8.00 to 494.92 h), and the median time from pacemaker cessation to its removal of 3 days (ranging from 2 to 9 days). All children who had the endocardial TP successfully removed had their heart rhythm restored to normal sinus rhythm at discharge, and they did not exhibit any of the clinical symptoms present at admission or any new symptoms, with satisfactory cardiac output. Among the children who transitioned to permanent pacemakers due to the failure to adjust or discontinue the endocardial TP, no complications such as iatrogenic infection or thrombosis were observed during the follow‐up period.

Like other retrospective studies, this study has its limitations. Although the data was sourced from a large tertiary children's medical center in Western China, the single‐center design might lead to bias. Therefore, to gain a more comprehensive understanding of the use of endocardial TP in the treatment of critical cardiovascular conditions in children, there is an urgent need for studies with larger samples sizes, multiple‐center collaboration, and extended follow‐up periods.

## 5 CONCLUSIONS

In summary, endocardial TP in children with FM and malignant arrhythmias has shown definite and significant therapeutic effects in critically ill patients. Additionally, no device‐related complications were observed during the limited follow‐up period. For children who fail to restore normal sinus rhythm after temporary pacing withdrawal or adjustment, consideration of permanent pacemaker implantation may be necessary, with long‐term follow‐up required to evaluate treatment outcomes. This study provides important clinical experience and insights into the management of pediatric cardiovascular diseases.

## AUTHOR CONTRIBUTIONS

Ping Xiang and Mi Li formulated the study plan and supervised the project; Zhongyuan Liu, Kaijun Zhang, Jun Ren, Penghui Yang collected data; Zhongyuan Liu and Kaijun Zhang drafted the manuscript and prepared the table; Ping Xiang critically commented on and revised the manuscript.

## CONFLICT OF INTEREST STATEMENT

The authors declare no conflicts of interest.

## ETHICS STATEMENT

In this study, all legal guardians of the children were fully informed about the potential risks and possible complications associated with the implantation of the transvenous pacemaker prior to the surgery and had signed informed consent forms. And no personal details, pictures or videos are included in this study. The study adhered to the principles of the Declaration of Helsinki (revised version 2018) and had been approved by the Ethics Committee of the Children's Hospital of Chongqing Medical University.

## Data Availability

The data that support the findings of this study are available on request from the corresponding author. The data are not publicly available due to privacy or ethical restrictions.
